# Synergistic combination of DT-13 and topotecan inhibits human gastric cancer via myosin IIA-induced endocytosis of EGF receptor *in vitro* and *in vivo*

**DOI:** 10.18632/oncotarget.8843

**Published:** 2016-04-20

**Authors:** Xiao-Wen Yu, Sensen Lin, Hong-Zhi Du, Ren-Ping Zhao, Shu-Yun Feng, Bo-Yang Yu, Lu-Yong Zhang, Rui-Ming Li, Chang-Min Qian, Xue-Jun Luo, Sheng-Tao Yuan, Li Sun

**Affiliations:** ^1^ Jiangsu Center for Pharmacodynamics Research and Evaluation, China Pharmaceutical University, Nanjing, China; ^2^ Jiangsu Key laboratory of Drug Screening, China Pharmaceutical University, Nanjing, China; ^3^ Department of Biophysics, University of Saarland, Homburg, Germany; ^4^ Jiangsu Key Laboratory of TCM Evaluation and Translational Research, Department of Complex Prescription of TCM, China Pharmaceutical University, Nanjing, China; ^5^ Tasly Research Institute, Tianjin Tasly Holding Group Co. Ltd., Tianjin, China

**Keywords:** DT-13, topotecan, NM IIA, EGFR, gastric cancer

## Abstract

Combination therapy has a higher success rate for many cancers compared to mono-therapy. The treatment of Topotecan (TPT) on gastric cancer (GC) is limited by its toxicity and the potential drug resistance. We found that the combination of the saponin monomer 13 from the dwarf lilyturf tuber (DT-13), performing anti-metastasis and anti-angiogenesis effects, with TPT synergistically induced apoptotic cytotoxicity in GCs with high EGF receptor (EGFR) expression, which was dependent on DT-13-induced endocytosis of EGFR. With TPT, DT-13 promoted EGFR ubiquitin--mediated degradation through myosin IIA-induced and Src/ caveolin-1 (Cav-1)-induced endocytosis of EGFR; inhibited EGFR downstream signalling and then increased the pro-apoptotic effects. Moreover, the synergistic pro-apoptotic efficacy of DT-13 and TPT in GCs with high EGFR expression was eliminated by both the NM II inhibitor (−)-blebbistatin and MYH-9 shRNA. The combination therapy of DT-13 with TPT showed stronger anti-tumour effects *in vivo* compared with their individual effects. Moreover, the results of combination therapy revealed selective upregulation of pro-apoptotic activity in TUNEL assays and cleaved caspase-3 and NM IIA in immunohischemical analysis; while specific downregulation of p-extracellular regulated kinase 1/2 (p-ERK1/2), EGFR and Cav-1 in immunohischemical analysis. Collectively, these findings have significant clinical implications for patients with tumours harbouring high EGFR expression due to the possible high sensitivity of this regimen.

## INTRODUCTION

Gastric cancer (GC) remains a major health issue and a leading cause of death worldwide. The incidence of GC worldwide has been decreasing in recent years, but it remains the fourth most common malignant tumour and performs the second highest mortality rate of the cancerous diseases [1]. GC patients treated with either surgery or chemoradiotherapy have a poor prognosis and low five-year survival rate. Recently, an increasing number of GC targets have been found, including EGFR, HER2 and VEGF [2]; several studies have demonstrated that the high expression of EGFR in GC has a close relationship with its occurrence, development and biological behaviour [3]. Moreover, Aichler M *et al*. reported that high EGFR expression induced chemoresistance and was correlated with a poor prognosis in the clinic [4, 5].

Fluorouracil, doxorubicin, cisplatin and many other drugs are in clinical trials for the treatment of GC. TPT, which is a semi-synthetic analogue of the new Topo-inhibitor camptothecin, has been licensed as an anti-cancer agent for small cell lung cancer [6] and ovarian cancer [7]. DT-13, a saponin of the dwarf lilyturf tuber *Ophiopogon japonicus* wall (Family: Convallariaceae), possesses anticancer activities against various types of cancers [8] and anti-angiogenesis activity [9] on multiple targets, such as Egr-1, VEGF, CCR-5, Hif-1α and MMP2/9 [10–12]. In a recent study research, DT-13 attenuated tumour necrosis factor-α-induced vascular inflammation that was associated with Src/NF-кB/MAPK pathway modulation [13]. Using column chromatography, we have verified that NM IIA was the specific target of DT-13. We have found TPT downregulated the expression of NM IIA, which would attenuate the effectiveness of the TPT therapy. As taking the advantage of combination therapies (i.e., avoiding the risk of the development of resistance, increasing the effectiveness of the therapy, and the effectiveness of clinical combination therapies with TPT), we designed DT-13 combined with TPT, to increase the expression of NM IIA and increase the effectiveness of TPT therapy for GCs.

NM II is an ATP-driven molecular motor that plays diverse roles in cell physiology. Through crosslinking and translocation of actin filaments by utilizing energy from ATP hydrolysis, NM II can inhibit cellular reshaping and movement and consequently depress cell migration, adhesion, polarity, and cytokinesis [14–16]. NM II is a hexamer composed of two pairs of light chains (20 kDa and 17 kDa) [17] and distinct heavy chain (II-A, II-B, or II-C) along with three prominent genes (MYH-9, MYH-10 and MYH-14) that encode the NMHC (non-muscle myosin heavy chain; 230 kDa) proteins [18]. NM IIA has the highest rate of ATP hydrolysis of the three NM II isoforms and propels actin filaments more rapidly than NM IIB and NM IIC [14, 19]. The different enzymatic and motor activities of the NM IIs reside in their N-terminal domain, while the C-terminal rod and non-helical tail determine the assembly of myosin filaments and the intracellular localization of the NM II isoforms [20].

Kim JH *et al*. reported that NM II was required for the endocytosis of EGFR and the modulation of the EGFR-dependent activation of downstream signals, including ERK1/2 and Akt [21]. Based on these findings, we developed the hypothesis that DT-13 might be able to correlate to NM II and synergistically combine with TPT to inhibit the high EGFR expression GCs through increasing NM IIA modulation of EGFR endocytosis and downstream signalling.

## RESULTS

### DT-13 combined with TPT had a synergistic anti-cancer effect on GCs with high EGFR expression

**Figure 1:**
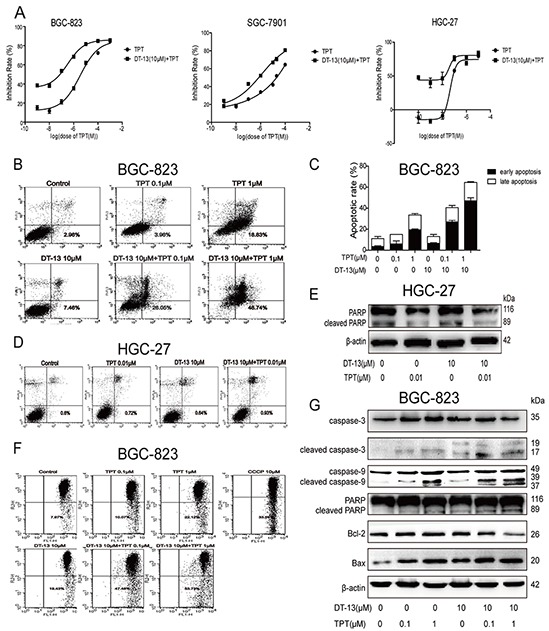
DT-13 combined with TPT promoted pro-apoptotic activity in BGC-823 cells **A.** The combination inhibitory effects of DT-13 combined with TPT on the survival of GCs (BGC-823, SGC-7901 and HGC-27) were measured by MTT assay after the combined treatment for 72 h. The early apoptosis was assessed by Annexin-V/PI staining in BGC-823 cells **B–C.** and HGC-27 cells **D.** after the cells were treated with the combined treatment for 48 h. **E.** The cleaved PARP protein level was assessed by western blotting analysis in HGC-27 cells. **F.** JC-1 staining was used to assess the apoptosis induced by the combined treatment in BGC-823 cells. **G.** The protein levels of apoptosis-related proteins were checked by western blotting analysis in BGC-823 cells.

Based on the potential of DT-13 and TPT on inhibiting the EGFR-involved pathway [22], we measured the inhibitory effect of DT-13 combined with TPT on the survival of GCs with different EGFR expression levels, such as: BGC-823 (high expression), SGC-7901 (high expression), and HGC-27 (low expression) cell lines (the EGFR level data were shown in Supplementary Figures S1A, S1B). After 72 h treatment, DT-13 combined with TPT exhibited a synergistic inhibitory effect on the survival of GCs with high EGFR expression (BGC-823 and SGC-7901) compared with TPT treatment alone. In contrast, no synergistic effect was observed in HGC-27 cells (Figure 1A). Moreover, the combined treatment changed the morphology of the high EGFR-expressing cells (Supplementary Figure S1C). These results indicated that DT-13 combined with TPT exerted a synergistic inhibitory effect on cell survival only in GCs with high EGFR expression.

### DT-13 combined with TPT promoted pro-apoptotic activity in BGC-823 cells

To investigate the mechanism of the combined treatment on the survival of the high EGFR-expressing GCs, we measured the effect of the combined treatment on pro-apoptotic activity. As shown, a stronger pro-apoptotic activity was observed in the combination treatment groups after 48 h of treatment compared with the single drug treatment groups (Figures 1B, 1C, Supplementary Figure S2A and Supplementary Figure S2B). A change in the nuclei was also observed after the combination treatment (Supplementary Figure S1D). In contrast, the combination treatment didn't exert pro-apoptotic activity in HGC-27 cells (low EGFR expression) (Figures 1D, 1E).

Mitochondria release caspase-activating proteins into the cytosol, thereby forming the apoptosome where caspases bind and become activated [23]. To confirm the pro-apoptotic effect of the combination treatment, we measured the ΔΨm in the cells via JC-1 staining and flow cytometry analysis. As shown in Figures 1F and Supplementary Figure S2C, the proportion of cells emitting green fluorescence (apoptosis) in the combination group was significantly higher compared to the single-drug groups. We also measured the levels of proteins involved in the apoptosis pathway. The combination treatment increased the expression of cleaved caspase-3, cleaved caspase-9, cleaved PARP, and Bax and reduced the expression of Bcl-2, ongoing induce apoptosis in the high EGFR-expressing GCs (Figures 1G, Supplementary Figure S2D). These findings clearly showed the mitochondria mediated apoptosis in GCs following treatment with the combination of DT-13 and TPT.

### DT-13 combined with TPT down-regulated EGFR and its downstream signalling

**Figure 2:**
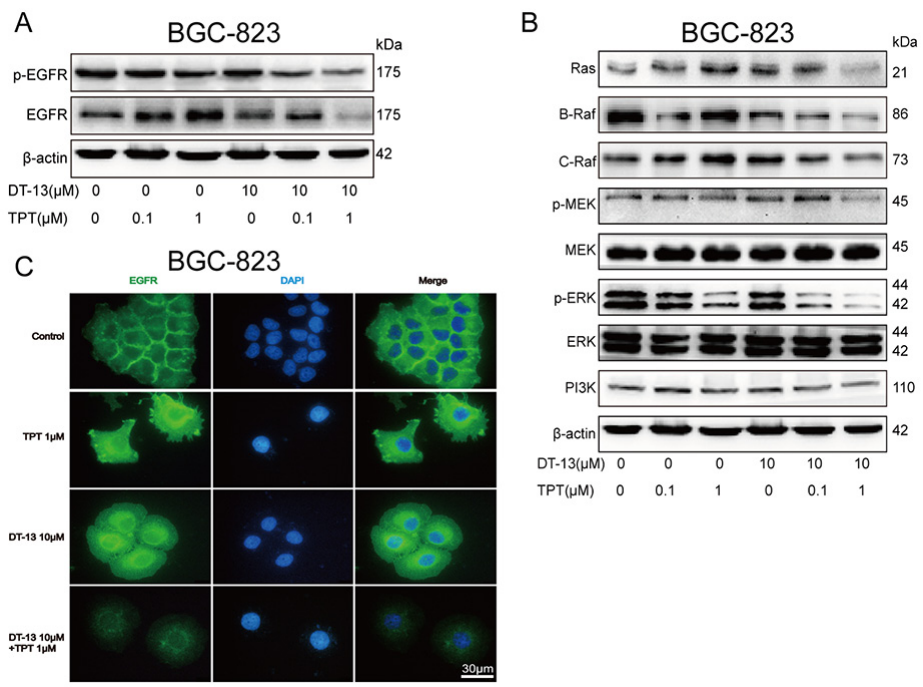
DT-13 combined with TPT down-regulated EGFR and its downstream signaling The cells were measured after the combined treatment for 48 h. **A.** Effects of DT-13 combined with TPT on EGFR total protein levels and phosphorylated protein levels were detected by western blotting analysis in BGC-823 cells. **B.** The combination treatment significantly reduced Ras/Raf/MEK/ERK1/2 pathway and PI3K expression in BGC-823 cells. **C.** The fluorescence intensity of EGFR was assessed in BGC-823 cells.

Due to the different effects of the combination treatment on GCs with different EGFR levels, we investigated the mechanism underlying the effect of the combination treatment on the EGFR pathway. In BGC-823 and SGC-7901 cells, the combination treatment not only reduced the p-EGFR level but also reduced total EGFR expression (Figures 2A, Supplementary Figure S2E). To confirm the regulation of the combination treatment on the EGFR pathway, we measured the downstream events of EGFR. The combination treatment exhibited a stronger inhibitory effect on Ras/Raf/MEK/ERK1/2 pathway and PI3K level than TPT or DT-13 alone in BGC-823 (Figure 2B) and SGC-7901 cells (Supplementary Figure S2G). In previous studies, the Ras/Raf/MEK/ERK1/2 and PI3K signalling cascades were shown to play critical roles in the transmission of signals from growth factor receptors to regulate gene expression and prevent apoptosis [24, 25]. Our findings indicate that the combined effect of DT-13 and TPT leads to GC apoptosis via EGFR and its downstream signalling.

Moreover, the combination treatment decreased not only EGFR expression but also EGFR distribution on cell plasm membrane. In the BGC-823 and SGC-7901 combination treatment group, we observed a significant decrease in the fluorescence intensity of EGFR both in throughout the cells and on the plasma membrane (Figures 2C, Supplementary Figure S3A). This result indicated that DT-13 combined with TPT exerts a pro-apoptotic activity via depressing EGFR and its downstream signalling.

### DT-13 promoted the degradation of EGFR through the induction of EGFR endocytosis by myosin IIA

**Figure 3:**
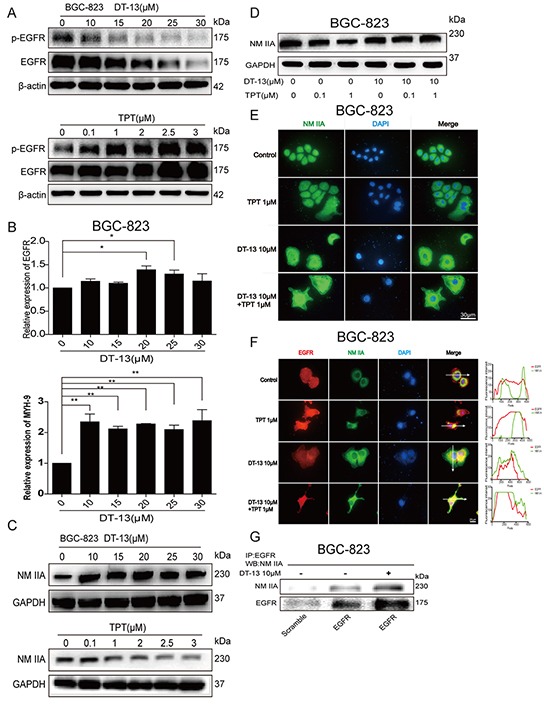
DT-13 could promote the degradation of EGFR through endocytosis of EGFR induced by myosin IIA **A.** The effect of DT-13 and TPT separately on EGFR and p-EGFR in BGC-823 cells. The cells were treated with DT-13 (10, 15, 20, 25, 30 μM), or TPT (0.1, 1, 2, 2.5, 3.5 μM) for 48 h, the total and phosphorylation levels of EGFR were checked by western blot analysis. **B.** The effect of DT-13 alone on EGFR and NM IIA mRNA expression in BGC-823 cells. The cells were treated with DT-13 (10, 15, 20, 25, 30 μM) for 48 h, and the mRNA levels of EGFR and NM IIA were determined. **C.** Western blot assays were used to examine the effect of DT-13 and TPT separately on NM IIA in BGC-823 cells after cells were treated with DT-13 (10, 15, 20, 25, 30 μM), or TPT (0.1, 1, 2, 2.5, 3.5 μM) for 48 h, GAPDH as internal reference. **D.** The expression of NM IIA in BGC-823 cells was checked by western blot assays after the combined treatment for 48 h. **E.** The immunofluorescence analysis was used to examine NM IIA in BGC-823 cells when DT-13 combined with TPT. **F.** The immunofluorescence localization method was used to verify the interaction of NM IIA with EGFR in BGC-823 cells. The location of the arrowed line is to measure the position of the fluorescence intensity. **G.** The co-immunoprecipitation assay was used to verify the interaction of NM IIA with EGFR in BGC-823 cells. Statistical analysis was performed using one-way ANOVA followed by Bonferroni's Multiple Comparison Test, *P< 0.05; **P< 0.01; for B statistical analysis was performed using at least three independent replicates.

The combination treatment reduced total and phosphorylated EGFR. However, it is not clear which drug (or both) owns that function. Thus, we measured the effect of DT-13 and TPT separately on EGFR and p-EGFR in BGC-823 and SGC-7901 cells. As shown in Figures 3A and Supplementary Figure S3B, a decrease in the expression of EGFR and p-EGFR was observed after the GCs were treated with DT-13 for 48 h; moreover, the reduction of EGFR was greater than the reduction of p-EGFR. Similarly, the expression of EGFR and p-EGFR was significantly increased after treatment of the GCs with TPT alone for 48 h. This result indicates that DT-13 may reduce p-EGFR via down-regulating the EGFR level. However, the EGFR mRNA level was not reduced after solo treatment with DT-13 (Figures 3B, Supplementary Figure S3C). Our findings indicated that DT-13 down-regulated the EGFR protein level via degradation of EGFR but not via regulating of the EGFR mRNA level.

Kim JH *et al*. reported that NM II was required for the endocytosis of EGFR [21]. Therefore, we focused on the potential relationship between EGFR and NM II in GCs after DT-13 treatment. Using column chromatography, we confirmed the affinity of DT-13 for NM IIA based on specific binding (data not shown). To reconfirm these findings for both drugs individually, we examined the effects of DT-13 and TPT alone or combination treatment on NM IIA. We found that the expression of NM IIA was increased by DT-13, whereas TPT suppressed the expression of NM IIA in BGC-823 (Figure 3C) and SGC-7901 cells (Supplementary Figure S3D). The combination treatment increased the NM IIA protein level in both cell lines (Figures 3D, Supplementary Figure S3E); the NM IIA mRNA level exhibited a same trend (Figures 3B, Supplementary Figure S3C).

The fluorescence intensity of NM IIA in BGC-823 and SGC-7901 cells was significantly increased in the combined group (Figures 3E, Supplementary Figure S4A); however, NM IIA distribution was not changed by the DT-13 single or combination treatment. To investigate a potential role for NM IIA in EGFR degradation, we measured the distribution of both NM IIA and EGFR after treatment. In the immunofluorescence analysis, DT-13 (alone or combination) not only decreased the fluorescence intensity of EGFR and increased NM IIA expression but also induced the colocalization of EGFR and NM IIA in the cells based on fluorescence intensity analysis by Image J (Figures 3F, Supplementary Figure S4B). However, the DT-13 alone and combination treatments did not change the distribution of NM IIA and EGFR in HGC-27 GCs (Supplementary Figure S4C). To confirm these results, we used the co-immunoprecipitation assay to determine the relationship between EGFR and NM IIA in the cytoplasm. The results showed that the EGFR protein level in the cytoplasm was increased by DT-13; the level of NM IIA binding with EGFR was also increased by DT-13 (Figures 3G, Supplementary Figure S4D). These data supported the hypothesis that DT-13 had the ability to promote EGFR degradation through myosin IIA-induced endocytosis of EGFR.

**Figure 4:**
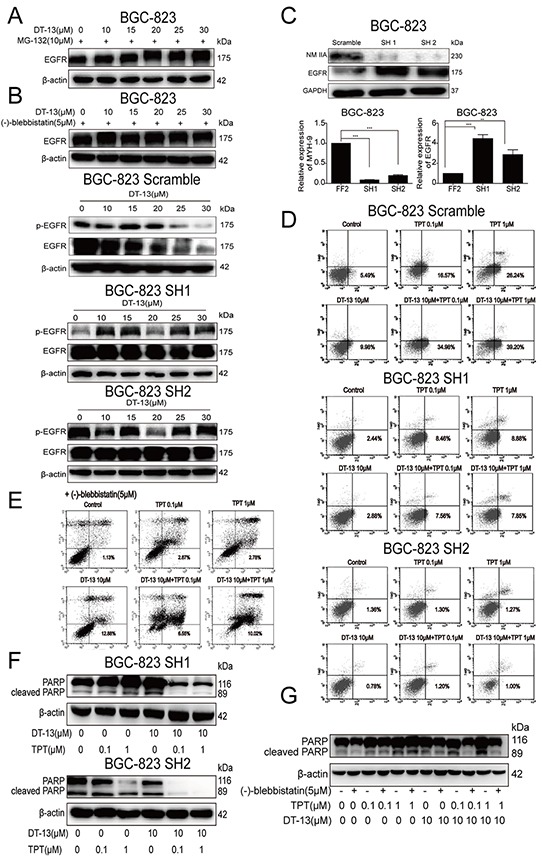
DT-13 increased NM IIA to contribute to the pro-apoptotic effect when combined with TPT **A.** To verify the expression of EGFR protein was ubiquitin mediated protein degradation, the cells were pro-treated with MG-132 for 2 h, and then added DT-13 (0, 10, 15, 20, 25, 30 μM) alone for 48 h. **B.** To verify the degradation of EGFR protein related to the activity of NM IIA, the cells were pre-treated with NM II inhibitor (−)-blebbistatin or knocked down NM IIA before DT-13 treatment. **C.** After NM IIA knock down, the protein and mRNA levels of EGFR and NM IIA were determined in BGC-823 cells. Annexin V/PI staining assay were used to determine the effects of both (−)-blebbistatin **D.** and the knock down of NM IIA **E.** on the apoptosis induced by the combination treatment. The level of cleaved PARP induced by the combination treatment was reduced by NM IIA knock down **F.** and (−)-blebbistatin (G) in BGC-823 cells. Statistical analysis was performed using one-way ANOVA followed by Bonferroni's Multiple Comparison Test, **P< 0.01; ***P< 0.001; for C statistical analysis was performed using at least three independent replicates.

To verify whether the expression of EGFR protein was decreased due to ubiquitin-triggered protein degradation, we pre-treated GCs with the proteasome inhibitor MG-132 for 2 h and then treated cells with DT-13 for 48 h. MG-132 significantly reversed the effect of DT-13 (alone or combination) on the EGFR protein reducing 48 h after DT-13 treatment (Figures 4A, Supplementary Figure S4E). Next, to examine whether the degradation of EGFR protein was correlated to NM IIA, we pre-treated the GCs with the NM II inhibitor (−)-blebbistatin or knocked down NM IIA. We found that both (−)-blebbistatin treatment and NM IIA knock-down reversed the effect of DT-13 (alone or combination) on the EGFR protein reducing (Figures 4B, Supplementary Figure S4F); indeed, the EGFR protein and mRNA levels were increased after NM IIA knock down (Figures 4C, Supplementary Figure S4G). There was no significant change observed on the mRNA levels of NM IIB and NM IIC (Supplementary Figure S4H). These findings showed that DT-13 promoted EGFR degradation through myosin IIA-induced endocytosis of EGFR.

### DT-13 increased NM IIA to contribute to the pro-apoptotic effect

Because EGFR was correlated to the pro-apoptotic effect, we investigated whether NM IIA was involved in the induction of apoptosis by DT-13. After NM IIA knock down, the effect of DT-13 (alone or combination) on proportion of early apoptosis was significantly reduced. Moreover, cleaved PARP was reduced by the NM IIA knock down when GCs (BGC-823 and SGC-7901) were treated with DT-13 (Figures 4D, 4F, Supplementary Figure S5A and Supplementary Figure S5C). Similar to NM IIA knock down, (−)-Blebbistatin also induced the reversal of early apoptosis and cleaved PARP level (Figures 4E, 4G and Supplementary Figure S5B). These data confirmed that DT-13 induced the endocytosis of EGFR via the up-regulation NM IIA, thereby resulting in GC apoptosis.

### Combinational pro-apoptotic effect was promoted by NM IIA

**Figure 5:**
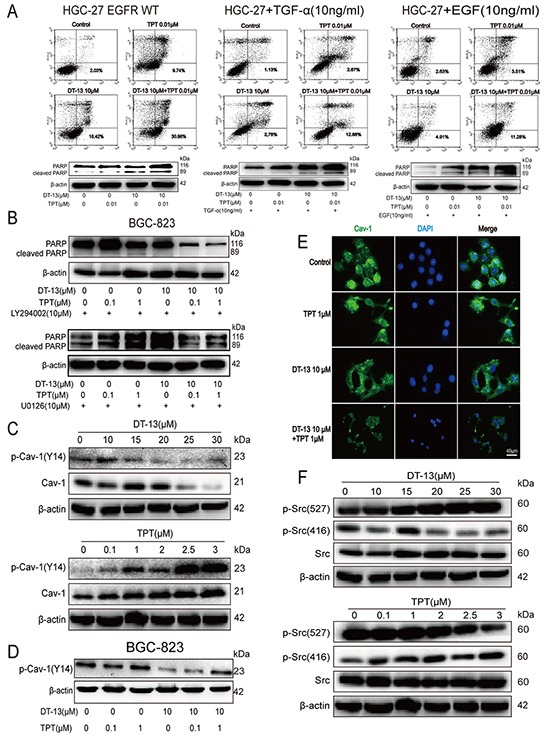
The effect of the combination treatment on the EGFR pathway and the Src/caveolin-1 pathway *in vitro* **A.** HGC-27 cells were transfected with the EGFR WT plasmid, or treated with EGF (10 ng/mL) or TGF-α (10 ng/mL), to up-regulate EGFR expression, the cleaved PARP and the proportion of cells undergoing early apoptosis was measured after the combined treatment. **B.** BGC-823 cells were treated with LY294002 (10 μM) or U0126 (10 μM) separately for 2 h before DT-13-TPT combination treatment, the cleaved PARP was determined by western blot analysis. **C.** The effect of DT-13 and TPT separately on Cav-1 and p-Cav-1 (Y14) in BGC-823 cells. The cells were treated with DT-13 (10, 15, 20, 25, 30 μM), or TPT (0.1, 1, 2, 2.5, 3.5 μM) for 48 h, the total and phosphorylation levels of Cav-1 were checked by western blot analysis. The cells were measured after the combined treatment for 48 h, the p-Cav-1 (Y14) was checked by western blot analysis in BGC-823 cells **D.** and the fluorescence intensity of Cav-1 was assessed in BGC-823 cells **E. F.** The effect of DT-13 and TPT separately on Src and p-Src in BGC-823 cells. The cells were treated with DT-13(10, 15, 20, 25, 30 μM), or TPT (0.1, 1, 2, 2.5, 3.5 μM) for 48 h, the total and phosphorylation levels of Src were determined by western blot analysis.

The above data showed that DT-13 combined with TPT had a stronger effect on GC inhibition and the induction of GC apoptosis (Figures 1B, 1E and 1F) compared with DT-13 or TPT single treatment. Moreover, our data showed that NM IIA was an important target of the induction of apoptosis in GCs with high EGFR expression by DT-13. Therefore, we focused on whether NM IIA was also a crucial target of the combination treatment. NM IIA knocking down and (−)-Blebbistatin can both reversed the apoptosis induced by the combination treatment (Figures 4D, Supplementary Figure S5C and 4E). Moreover, the level of cleaved PARP induced by the combination treatment was reduced by NM IIA knock down and (−)-blebbistatin, with the NM IIA knock down nearly eliminating all of the cleaved PARP in GCs treated with the combination treatment (Figures 5F, 5G, S5A and S5B). The data indicated that the effect of the combination treatment on GC apoptosis depended on the function of NM IIA in GCs with high EGFR expression.

To confirm the role of NM IIA in cells administered the combination treatment, we determined the effect of TPT on the EGFR pathway. The western blot results showed that TPT reduced NM IIA expression (Figure 3C) and increased EGFR expression (Figure 3A). Similar results were observed in the immunofluorescence analysis (Figures 3E, 2C). However, when TPT was combined with DT-13, the effect of TPT on NM IIA/EGFR was reversed by DT-13 (Figures 3D, 2A).

To confirm the effect of the combination treatment on the EGFR pathway, we determined the effect of the combination treatment on the apoptosis of HGC-27 cells transfected with the EGFR wild-type plasmid. The above data showed that DT-13-TPT combination did not exert a synergistic effect in HGC-27 cells with the low level of EGFR expression. However, when HGC-27 cells were transfected with the EGFR WT plasmid (Supplementary Figure S5D) or treated with EGF or TGF-α to up-regulate EGFR expression (Supplementary Figure S5E), the proportion of cells undergoing early apoptosis in DT-13-TPT combination treatment group was increased and the cleaved PARP protein level was up-regulated (Figure 5A). Similarly, when BGC-823 cells with higher EGFR expression were pre-treated with 10 μM MEK inhibitor (U0126) or 10 μM PI3K inhibitor (LY294002) for 2 h before treatment with DT-13-TPT combination, the effect of DT-13-TPT combination on pro-apoptotic activity was inhibited (Figure 5B). We also found EGFR depletion using siRNA or EGFR inhibitor (Erlotinib) exhibit similar effect in combined with TPT (Supplementary Figure S6A, S6B, S6C, S6D), but the ability of both in increasing the effectiveness of TPT therapy was inferior to DT-13. These findings supported that EGFR signaling axis plays a key role in sensitizing gastric cancer cells with high EGFR expression to TPT treatment.

### Effect of DT-13 combined with TPT on Src/Cav-1

Various receptor and non-receptor kinases such as the EGFR are localized in the Caveolae. Cav-1 binds to EGFR and activates multiple signalling networks [26–28]. Peculiar Src expression and kinase activity have been found in a number of different tumours, including GCs; these changes in expression could upregulate tyrosine phosphorylation of Cav-1 [29, 30] and inhibit the effects of the cell membrane on EGFR endocytosis [31]. Preclinical experiments have demonstrated that Src kinase activity may activate EGFR activity [32]. Cav-1 and p-Cav-1(Y14) were reduced when BGC-823 cells were treated with DT-13; however, the TPT-treated group had the opposite result (Figure 5C). When DT-13 was combined with TPT, the increased p-Cav-1(Y14) level was reduced (Figure 5D). Using immunofluorescence, we determined that the fluorescence intensity of Cav-1 was faded in the combination group (Figure 5E). Furthermore, DT-13 inhibited Tyr527 dephosphorylation and Tyr416 phosphorylation and downregulated Src activity; TPT up-regulated Src activity via promoting Tyr527 dephosphorylation and Tyr416 phosphorylation (Figure 5F). These results suggested that DT-13 could accelerate the endocytosis of EGFR through the down-regulation of Src activity, thereby reducing the level of p-Cav-1(Y14). When DT-13 was combined with TPT, DT-13 could eliminate the side effects of TPT.

### Combination treatment and inhibition of tumour growth in BGC-823-xenografted nude mice

**Figure 6:**
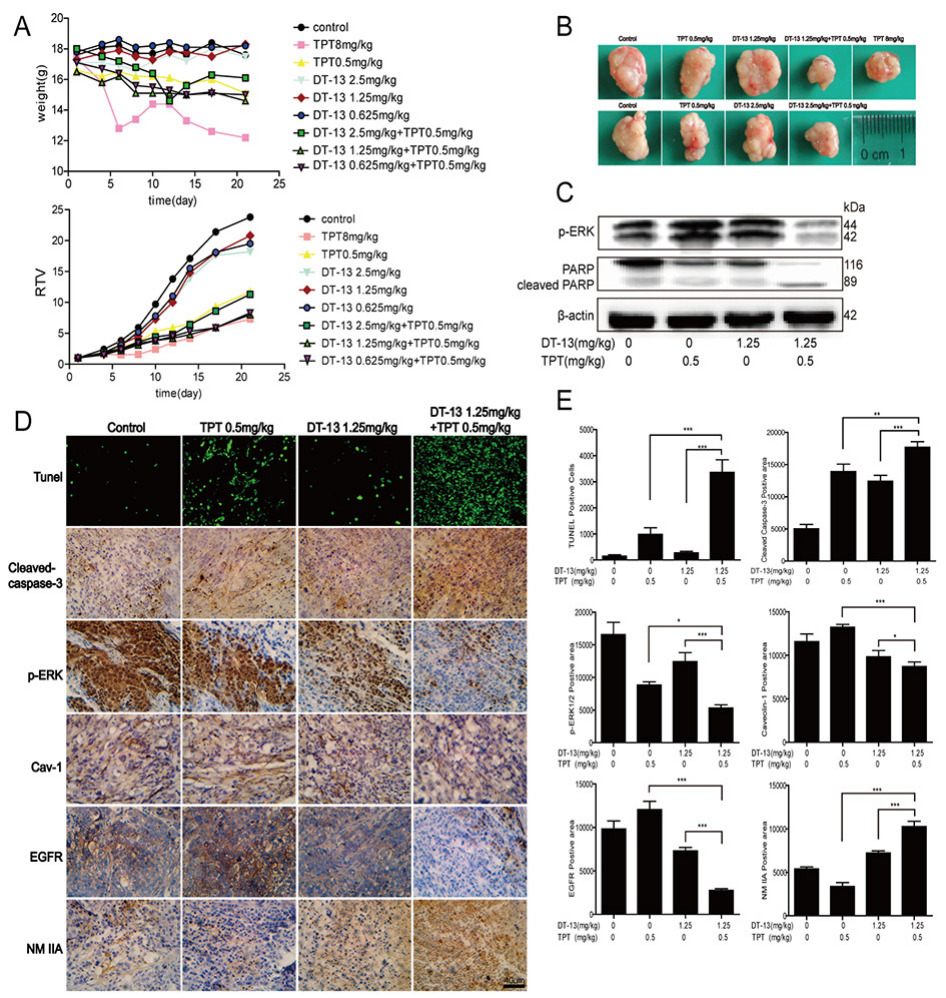
Combination treatment and inhibition of tumor growth in BGC-823 xenograft nude mice **A.** The animal weight and RTV were examined *in vivo.* **B.** Representative photographs of tumors respectively, in each group at the end of experiment. **C.** The cleaved PARP and p-ERK1/2 were checked by western blot analysis in DT-13-TPT combination-treated group. **D-E.** Tumor tissues from BGC-823-xenografted nude mice after DT-13-TPT combination treatment were analyzed by TUNEL assays, and immunohistochemistry staining of cleaved caspase-3, p-ERK, Cav-1, EGFR and NM IIA. Statistical analysis was performed using one-way ANOVA followed by Bonferroni's Multiple Comparison Test, *P< 0.05; **P< 0.01; ***P< 0.001.

DT-13 combined with TPT was not only effective *in vitro* but also acted as an effective anti-cancer regimen *in vivo*. We evaluated the effect of DT-13-TPT combination *in vivo* using an established BGC-823 cell xenograft model. As shown in Figures 6A and 6B, 1.25 mg/kg DT-13 combined with 0.5 mg/kg TPT exerted a significant synergistic inhibitory effect on BGC-823 xenografts. In tumour tissues from the BGC-823-xenografted nude mice treated with DT-13-TPT combination, the positive areas for TUNEL, cleaved caspase-3 and NM IIA were increased, while the positive areas for p-ERK1/2, EGFR and Cav-1 were reduced (Figures 6D, 6E). Additionally, more EGFR appeared in the cytoplasm in cells from DT-13-TPT combination-treated group; the protein levels of cleaved PARP and p-ERK1/2 were increased in DT-13-TPT combination-treated group (Figure 6C). These results showed that DT-13 combined with TPT boosted the pro-apoptotic effect via EGFR downstream signalling and the Cav-1 pathway, which was consistent with the *in vitro* data.

## DISCUSSION

Combination treatments are frequently used to treat different cancers to increase the curative effect and avoid high doses and toxic side effects; combination therapy may also help to avoid the development of drug resistance. Therefore, the development of a reasonable and efficient combination strategy for the effective chemotherapy of tumours is of great significance. Combining different agents can be more effective (additive or synergistic), because multiple pathways can be targeted [33, 34]. However, previous studies have suggested that the benefits of combination treatment are selective and that only specific subcategories of cancers are exceptionally sensitive. Okabe T *et al.* demonstrated that the synergistic effects of gefitinib and 5-fluorouracil combined treatment was only observed in EGFR mutant (exon 19 deletion) NSCLC cell lines with MET amplification but not in cell lines with an additional EGFR T790M mutation [35]. According to these observations, it is likely that specific drug combinations are mostly effective against a specific phenotypic and genotypic subcategory of cancer.

In this study, we observed that BGC-823 and SGC-7901 cells (with higher EGFR levels) responded best to low dose DT-13-TPT combination treatment. In contrast, HGC-27 cells (with lower EGFR levels) treated with DT-13-TPT combination showed minimal cell apoptosis. When HGC-27 cells were induced to overexpress EGFR, the proportion of cells in early apoptosis following DT-13-TPT combination treatment was increased and the cleaved PARP protein level was upregulated. These findings led us to conclude that EGFR signalling axis plays a key role in sensitizing GCs with high EGFR expression to low dose DT-13-TPT combination treatment. Compelling evidence indicated that the downstream signalling molecules and pathways, B-Raf and C-Raf, which is activated by forming heterodimer in physiological progression. Additionally, the specific inhibitor of B-Raf could activate C-Raf and it regulated signalling, in a B-Raf independent manner [36], suggesting that the downstream signalling pathway activities is inhibited on the condition that completely inhibiting the activation of formed heterodimer [37]. In our observation, the TPT mono-therapy sufficiently reduced the B-Raf level but not C-Raf (Figures 2B, S2F, S2G), which results in partial downregulation on MEK/ERK signalling; while the TPT and DT-13 comb-therapy significantly decreased both B-Raf and C-Raf to markedly depress the MEK/ERK signalling, eventually promoted the pro-apoptotic activity more efficiency compared with TPT or DT-13 mono-therapy.

**Figure 7:**
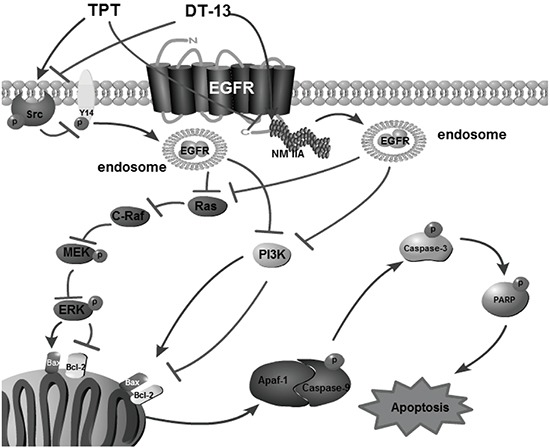
A schematic representation of a hypothesized mechanism of the synergistic combination of DT-13 and TPT inhibits human gastric cancer via myosin IIA-induced endocytosis of EGFR

This study described the use of DT-13-TPT combination to induce pro-apoptotic effect and killing cancer cells. We found that the addition of DT-13 with TPT in BGC-823 cells reduced the IC_50_ 50-fold as TPT. Upon further investigation, we found two ways by which DT-13 might expedite EGFR endocytosis and hinder its key role in the initiation of signalling response. First, DT-13 had the ability to increase NM IIA expression and might promote NM IIA binding to EGFR, thereby expediting the endocytosis of EGFR as described in previous studies [21]. Second, DT-13 was involved in the promotion of the endocytosis of EGFR through the down-regulation of Src protein activity, leading to the suppression of p-Cav-1 (Y14) and the enhanced endocytosis ability of Caveolae for EGFR [26, 38, 39] (Figure 7).

Ligand-induced endocytosis of EGFR is an important process for the regulation of signal transduction, cellular dynamics, and cell-cell communication. Caveolae is a raft-like structure that belongs to the route of endocytosis of EGFR doesn't depend on the clathrin pathway, which was characterized by its insensitivity to the functional ablation of clathrin, a fact that led to its rather non-descriptive definition of non-clathrin endocytosis (NCE) [40–42]. The requirement of NM IIA for EGFR endocytosis was first reported by Kim JH.et al [21] and wasn't further explored in depth until now. We are the first to report that the degradation of EGFR is induced by NM IIA, causing EGFR endocytosis. Despite the importance of EGFR in GC development in the clinic [43], the binding of NM IIA and EGFR had not been applied to drug research. Our present study verified the interaction of NM II with EGFR and indicated that this novel mechanism was a target for combination drugs; this is a novel aspect of our findings that has not been previously reported.

NM IIA has been reported to regulate cell migration, adhesion, polarity, and cytokinesis [14–16]; however, the correlation between NM IIA and cell apoptosis has not been reported previously. Our findings highlight for the first time this correlation of NM IIA with cancer cell apoptosis by using the NM II inhibitor and MYH-9 shRNA. These findings showed that the percentage of GCs in early apoptosis was reduced when NM IIA was blocked or the activity of NM IIA was inhibited; we also postulated that the NM IIA mediated EGFR degradation process is related to the expression level of EGFR. For instance, the NM IIA mediated EGFR degradation process was only activated when EGFR expression reached a threshold, or conversely. In this research, DT-13 monotherapy increased the activation of NM IIA but fail to activate the NM IIA mediated EGFR degradation process due the expression of EGFR is below the threshold; however, Our research group will be investigating the whole picture of the contextual pathways between the NM IIA mediated EGFR degradation process and EGFR expression level, and these findings may need to be explored in depth to evaluate the relevancy of EGFR expression and the effects of DT-13 on the NM IIA promoter regions.

## MATERIALS AND METHODS

### Cell culture and plasmids

Human gastric cancer (BGC-823, SGC-7901, HGC-27, NCI-N87 and MGC-803) cells and embryonic kidney 293T cells were purchased from The Shanghai Institute of Life Science, Chinese Academy of Sciences. BGC-823 cells were maintained in DMEM with 10% fetal bovine serum (FBS, Gibco) and antibiotics (100 units/ml penicillin and 100 mg/ml streptomycin). 293T cells were cultured in DMEM/F12 with 10% FBS and antibiotics (100 units/ml penicillin and 100 mg/ml streptomycin). Other cells were grown in RMPI-1640 with 10% FBS and antibiotics (100 units/ml penicillin and 100 mg/ml streptomycin). The cells were incubated in a humidified atmosphere with 5% CO_2_ at 37°C. The MYH-9 lentivirus shRNA was purchased from Shanghai GenePharma Co., Ltd. The EGFR plasmid was obtained from Beijing Zhongyuan Ltd. Cell transfection and plasmid construction are described in the Supplementary Methods.

### Cell growth inhibition assay

Cells were seeded into 96-well plates in 180 μL of medium and incubated for 12 h. Then, the cells were cultured in the presence of DT-13 and TPT alone or in combination for 72 h. Afterwards, 5 mg/mL of MTT solution (20 μL/well) was added and the cells were cultured in a 5% CO_2_ incubator at 37°C for 4 h; then, the supernatant was discarded and DMSO was added (150 μL/well). The suspension was placed on a micro-vibrator for 5 min, and the absorbance was measured at 570 nm using a Universal Microplate Reader (EL800; Bio-tek Instruments Inc.). The experiments were performed in triplicate in a parallel manner for each concentration, and the results are presented as the mean ± SD. The inhibitory ratio was calculated by the following formula: inhibitory ratio (%) = (1–average absorbance of treated group/average absorbance of control group) × 100.

### Annexin V/PI double staining

After treatment 48 h, apoptotic cells were identified by the Annexin V-FITC Apoptosis Detection kit (BD Biosciences) in accordance with the manufacturer's instructions. Flow cytometric analysis was performed immediately after supravital staining. Data acquisition and analysis were performed in a Becton Dickinson FACS-Calibur flow cytometer using the Cell Quest software (Franklin Lakes).

### Detection of changes in mitochondrial membrane potential (ΔΨm) by JC-1

Mitochondrial membrane potential (ΔΨm) was evaluated using JC-1 (Thermo Fisher Scientific) staining and flow cytometry analysis. BGC-823 and SGC-7901 cells were treated with 10 μM DT-13 with or without various concentrations of TPT for 48 h. JC-1 detection was performed according to the manufacturer's protocol.

### Co-immunoprecipitation and immunofluorescence assay

Co-immunoprecipitation of EGFR with NM IIA and their co-localization in GCs are described in Supplementary Methods.

### Western blot analysis

After treatment 48 h, cellular proteins were extracted and Western blot analysis was performed as previously described [9]. All primary antibodies were purchased from Cell Signalling Technology, Inc (Cell Signaling Technology). Horseradish peroxidase (HRP)-conjugated anti-mouse immunoglobulin G (Sigma-Aldrich) and anti-rabbit immunoglobulin G (Cell Signaling Technology) were used as the secondary antibodies. Protein bands were visualized using enhanced chemiluminescence reagents (Millipore).

### Quantitative real-time PCR

Total cellular RNA was isolated with the TRIzol® Reagent (Vazyme) and reverse transcribed with the RevertAid^TM^ First Strand cDNA Synthesis Kit (Takara). The mRNA level was measured with the SYBR Green master mix (Vazyme). The amount of mRNA for each gene was standardized with the internal control (18s mRNA). Each treatment group was compared with the control group to show the relative mRNA level. The primer sequences for quantitative RT-PCR are provided in Supplementary Table S1.

### In vivo tumorigenicity

Female athymic BALB/c nude mice (5–6 weeks old) with body masses ranging from 18 to 22 g were supplied by the Shanghai Institute of Materia Medica, Chinese Academy of Sciences. For subcutaneous (S.C) injection of gastric cancer cells, sub-confluent BGC-823 cells were collected in serum-free medium (10^6^ cells/100 μL). Then, the cell suspension was injected subcutaneously into mice in one flank (n= 6). All assays were repeated at least three times. Animal care and surgery protocols were approved by the Animal Care Committee of China Pharmaceutical University. All animals were treated appropriately and used in a scientifically valid and ethical manner.

### Immunohistochemical analysis

Immunohistochemical analysis for apoptosis, cleaved-caspase 3, and p-ERK, Cav-1, EGFR and NM IIA in tumor tissues are described in Supplementary Methods.

### Statistical analysis

All of the results were presented as the mean ± SD from triplicate experiments performed in a parallel manner unless otherwise indicated. Statistically significant differences (One-way ANOVAs followed by Bonferroni's Multiple Comparison Test) were determined using GraphPad Prism 6 software. A value of P< 0.05 was considered significant, and values of P< 0.01 and P< 0.001 were considered highly significant.

## SUPPLEMENTARY METHODS, FIGURES AND TABLE



## References

[1] Deng JY, Liang H (2014). Clinical significance of lymph node metastasis in gastric cancer. World J Gastroentero : WJG.

[2] Cristescu R, Lee J, Nebozhyn M, Kim KM, Ting JC, Wong SS, Liu J, Yue YG, Wang J, Yu K, Ye XS, Do IG, Liu S, Gong L, Fu J, Jin JG (2015). Molecular analysis of gastric cancer identifies subtypes associated with distinct clinical outcomes. Nat Med.

[3] Zhang L, Yang J, Cai J, Song X, Deng J, Huang X, Chen D, Yang M, Wery JP, Li S, Wu A, Li Z, Liu Y, Chen Y, Li Q, Ji J (2013). A subset of gastric cancers with EGFR amplification and overexpression respond to cetuximab therapy. Sci Rep-UK.

[4] Aichler M, Motschmann M, Jutting U, Luber B, Becker K, Ott K, Lordick F, Langer R, Feith M, Siewert JR, Walch A (2014). Epidermal growth factor receptor (EGFR) is an independent adverse prognostic factor in esophageal adenocarcinoma patients treated with cisplatin-based neoadjuvant chemotherapy. Oncotarget.

[5] Wheler J, Falchook G, Tsimberidou AM, Hong D, Naing A, Piha-Paul S, Chen SS, Heymach J, Fu S, Stephen B, Fok JY, Janku F, Kurzrock R (2013). Revisiting clinical trials using EGFR inhibitor-based regimens in patients with advanced non-small cell lung cancer: a retrospective analysis of an MD Anderson Cancer Center phase I population. Oncotarget.

[6] von Pawel J, Gatzemeier U, Pujol JL, Moreau L, Bildat S, Ranson M, Richardson G, Steppert C, Riviere A, Camlett I, Lane S, Ross G (2001). Phase ii comparator study of oral versus intravenous topotecan in patients with chemosensitive small-cell lung cancer. J Clin Oncol.

[7] Rodriguez M, Rose PG (2001). Improved therapeutic index of lower dose topotecan chemotherapy in recurrent ovarian cancer. Gynecol Oncol.

[8] Mimaki Y, Takaashi Y, Kuroda M, Sashida Y, Nikaido T (1996). Steroidal saponins from Nolina recurvata stems and their inhibitory activity on cyclic AMP phosphodiesterase. Phytochemistry.

[9] Zhao R, Sun L, Lin S, Bai X, Yu B, Yuan S, Zhang L (2013). The saponin monomer of dwarf lilyturf tuber, DT-13, inhibits angiogenesis under hypoxia and normoxia via multi-targeting activity. Oncol Rep.

[10] Sun L, Lin S, Zhao R, Yu B, Yuan S, Zhang L (2010). The saponin monomer of dwarf lilyturf tuber, DT-13, reduces human breast cancer cell adhesion and migration during hypoxia via regulation of tissue factor. Biol Pharm Bull.

[11] Ren-Ping Z, Sen-Sen L, Yuan ST, Yu BY, Bai XS, Sun L, Zhang LY (2014). DT-13, a saponin of dwarf lilyturf tuber, exhibits anti-cancer activity by down-regulating C-C chemokine receptor type 5 and vascular endothelial growth factor in MDA-MB-435 cells. Chin J Nat Med.

[12] Zhang Y, Liu J, Kou J, Yu J, Yu B (2012). DT-13 suppresses MDA-MB-435 cell adhesion and invasion by inhibiting MMP-2/9 via the p38 MAPK pathway. Mol Med Rep.

[13] Zhang Y, Sun M, Han Y, Zhai K, Tang Y, Qin X, Cao Z, Yu B, Kou J (2015). The saponin DT-13 Attenuates Tumor Necrosis Factor-alpha-induced Vascular Inflammation Associated with Src/NF-small ka, CyrillicB/MAPK Pathway Modulation. Int J Biol Sci.

[14] Vicente-Manzanares M, Ma X, Adelstein RS, Horwitz AR (2009). Non-muscle myosin II takes centre stage in cell adhesion and migration. Nat Rev Mol Cell Bio.

[15] Even-Ram S, Doyle AD, Conti MA, Matsumoto K, Adelstein RS, Yamada KM (2007). Myosin IIA regulates cell motility and actomyosin-microtubule crosstalk. Nat Cell Biol.

[16] Wilson CA, Tsuchida MA, Allen GM, Barnhart EL, Applegate KT, Yam PT, Ji L, Keren K, Danuser G, Theriot JA (2010). Myosin II contributes to cell-scale actin network treadmilling through network disassembly. Nature.

[17] Park I, Han C, Jin S, Lee B, Choi H, Kwon JT, Kim D, Kim J, Lifirsu E, Park WJ, Park ZY, Kim do H, Cho C (2011). Myosin regulatory light chains are required to maintain the stability of myosin II and cellular integrity. Biochem J.

[18] Golomb E, Ma X, Jana SS, Preston YA, Kawamoto S, Shoham NG, Goldin E, Conti MA, Sellers JR, Adelstein RS (2004). Identification and characterization of nonmuscle myosin II-C, a new member of the myosin II family. J Biol Chem.

[19] Kim KY, Kovacs M, Kawamoto S, Sellers JR, Adelstein RS (2005). Disease-associated mutations and alternative splicing alter the enzymatic and motile activity of nonmuscle myosins II-B and II-C. J Biol Chem.

[20] Nakasawa T, Takahashi M, Matsuzawa F, Aikawa S, Togashi Y, Saitoh T, Yamagishi A, Yazawa M (2005). Critical regions for assembly of vertebrate nonmuscle myosin II. Biochemistry-US.

[21] Kim JH, Wang A, Conti MA, Adelstein RS (2012). Nonmuscle myosin II is required for internalization of the epidermal growth factor receptor and modulation of downstream signaling. J Biol Chem.

[22] Zuco V, Supino R, Favini E, Tortoreto M, Cincinelli R, Croce AC, Bucci F, Pisano C, Zunino F (2010). Efficacy of ST1968 (namitecan) on a topotecan-resistant squamous cell carcinoma. Biochem Pharmacol.

[23] Bao Q, Shi Y (2007). Apoptosome: a platform for the activation of initiator caspases. Cell Death Differ.

[24] McCubrey JA, Steelman LS, Chappell WH, Abrams SL, Franklin RA, Montalto G, Cervello M, Libra M, Candido S, Malaponte G, Mazzarino MC, Fagone P, Nicoletti F (2012). Ras/Raf/MEK/ERK and PI3K/PTEN/Akt/mTOR cascade inhibitors: how mutations can result in therapy resistance and how to overcome resistance. Oncotarget.

[25] Haydn JM, Hufnagel A, Grimm J, Maurus K, Schartl M, Meierjohann S (2014). The MAPK pathway as an apoptosis enhancer in melanoma. Oncotarget.

[26] Abulrob A, Giuseppin S, Andrade MF, McDermid A, Moreno M, Stanimirovic D (2004). Interactions of EGFR and caveolin-1 in human glioblastoma cells: evidence that tyrosine phosphorylation regulates EGFR association with caveolae. Oncogene.

[27] Agelaki S, Spiliotaki M, Markomanolaki H, Kallergi G, Mavroudis D, Georgoulias V, Stournaras C (2009). Caveolin-1 regulates EGFR signaling in MCF-7 breast cancer cells and enhances gefitinib-induced tumor cell inhibition. Cancer Biol Ther.

[28] Chatterjee M, Ben-Josef E, Thomas DG, Morgan MA, Zalupski MM, Khan G, Andrew Robinson C, Griffith KA, Chen CS, Ludwig T, Bekaii-Saab T, Chakravarti A, Williams TM (2015). Caveolin-1 is Associated with Tumor Progression and Confers a Multi-Modality Resistance Phenotype in Pancreatic Cancer. Sci Rep-UK.

[29] Thomas S, Overdevest JB, Nitz MD, Williams PD, Owens CR, Sanchez-Carbayo M, Frierson HF, Schwartz MA, Theodorescu D (2011). Src and caveolin-1 reciprocally regulate metastasis via a common downstream signaling pathway in bladder cancer. Cancer Res.

[30] Joshi B, Strugnell SS, Goetz JG, Kojic LD, Cox ME, Griffith OL, Chan SK, Jones SJ, Leung SP, Masoudi H, Leung S, Wiseman SM, Nabi IR (2008). Phosphorylated caveolin-1 regulates Rho/ROCK-dependent focal adhesion dynamics and tumor cell migration and invasion. Cancer Res.

[31] Chang CC, Yang MH, Lin BR, Chen ST, Pan SH, Hsiao M, Lai TC, Lin SK, Jeng YM, Chu CY, Chen RH, Yang PC, Chin YE, Kuo ML (2013). CCN2 inhibits lung cancer metastasis through promoting DAPK-dependent anoikis and inducing EGFR degradation. Cell Death Differ.

[32] Wu W, Wages PA, Devlin RB, Diaz-Sanchez D, Peden DB, Samet JM (2015). SRC-mediated EGF receptor activation regulates ozone-induced interleukin 8 expression in human bronchial epithelial cells. Environ Health Persp.

[33] Zwitter M, Rajer M, Kovac V, Kern I, Vrankar M, Smrdel U (2011). Intermittent chemotherapy and erlotinib for nonsmokers or light smokers with advanced adenocarcinoma of the lung: a phase II clinical trial. J Biomed Biotechnol.

[34] Zhou Y, Liang C, Xue F, Chen W, Zhi X, Feng X, Bai X, Liang T (2015). Salinomycin decreases doxorubicin resistance in hepatocellular carcinoma cells by inhibiting the beta-catenin/TCF complex association via FOXO3a activation. Oncotarget.

[35] Okabe T, Okamoto I, Tsukioka S, Uchida J, Hatashita E, Yamada Y, Yoshida T, Nishio K, Fukuoka M, Janne PA, Nakagawa K (2009). Addition of S-1 to the epidermal growth factor receptor inhibitor gefitinib overcomes gefitinib resistance in non-small cell lung cancer cell lines with MET amplification. Clin Cancer Res.

[36] Khazak V, Astsaturov I, Serebriiskii IG, Golemis EA (2007). Selective Raf inhibition in cancer therapy. Expert opin ther tar.

[37] Xu TR, Vyshemirsky V, Gormand A, von Kriegsheim A, Girolami M, Baillie GS, Ketley D, Dunlop AJ, Milligan G, Houslay MD, Kolch W (2010). Inferring signaling pathway topologies from multiple perturbation measurements of specific biochemical species. Sci signal.

[38] Henriksen L, Grandal MV, Knudsen SL, van Deurs B, Grovdal LM (2013). Internalization mechanisms of the epidermal growth factor receptor after activation with different ligands. PloS one.

[39] Wilde A, Beattie EC, Lem L, Riethof DA, Liu SH, Mobley WC, Soriano P, Brodsky FM (1999). EGF receptor signaling stimulates SRC kinase phosphorylation of clathrin, influencing clathrin redistribution and EGF uptake. Cell.

[40] Sigismund S, Argenzio E, Tosoni D, Cavallaro E, Polo S, Di Fiore PP (2008). Clathrin-mediated internalization is essential for sustained EGFR signaling but dispensable for degradation. Dev Cell.

[41] Maldonado-Baez L, Wendland B (2006). Endocytic adaptors: recruiters, coordinators and regulators. Trends Cell Biol.

[42] Mayor S, Pagano RE (2007). Pathways of clathrin-independent endocytosis. Nat Rev Mol Cell Bio.

[43] Terashima M, Kitada K, Ochiai A, Ichikawa W, Kurahashi I, Sakuramoto S, Katai H, Sano T, Imamura H, Sasako M (2012). Impact of expression of human epidermal growth factor receptors EGFR and ERBB2 on survival in stage II/III gastric cancer. Clin Cancer Res.

