# Supplementary material for: Synergistic combination of DT-13 and topotecan inhibits human gastric cancer via myosin IIA-induced endocytosis of EGF receptor in vitro and in vivo
Source: Oncotarget. 2016 Apr 20;7(22):32990–3003. doi: 10.18632/oncotarget.8843 (PMC5078069; doi:10.18632/oncotarget.8843)
Supplement: Supplementary file 1 [file oncotarget-07-32990-s001.pdf]

SUPPLEMENTARY METHODS

Cell transfection and plasmid construction

The cells were seeded at a density of 5×10^4/well in a 24-well plate 24 h before transfection to achieve more than 70% confluence. 20 μL MYH-9 lentivirus shRNA and 20 μL scrambled sequence lentivirus shRNA were added into 2 mL fresh medium individually, and then added 2μL polybrene (Santa Cruz) after 24 h-treatment, lentivirus medium was replaced by fresh medium. The plate was incubated at 37°C for 48–72 h until the transfection efficiency was more than 80% and was then used in the experiments described below.

293T cells were seeded in dish (100× 20 mm) 24 h before transfection to achieve more than 70% confluence. We added 6 μL Fugene 6 (Promega) to 400 μL serum-free medium for 5 min, and then added psPAX2 packaging plasmid 3 μg, pMD2.G envelope plasmid 1 μg, and EGFR WT plasmid 4 μg for 30 min. After mixed thoroughly, we changed the medium of 293T with this medium for 24 h, and then we changed normal 13 mL for 24 h, meanwhile HGC-27 cell was seed in dish. We collected medium after 48 h, and centrifuged at 2000×rpm for 10 min, and then we added 10 mL supernatant and 10 μL polybrene to HGC-27 cell. The dish was incubated at 37°C for 48–72 h until the transfection efficiency was more than 80% and was then used in the experiments described below.

Co-immunoprecipitation assay

EGFR (Santa Cruz) was immunocaptured from cells extracts using polyclonal antibodies to EGFR cross-linked to protein G-agarose beads (Thermo Fisher Scientific). The immune-complexes were analyzed by western blotting and probed with antibody against NM IIA (Cell Signaling Technology).

Immunofluorescence assay

The cells were fixed with 4% paraformaldehyde in PBS at 30 min intervals, permeabilized with 0.5% Triton X-100, and blocked with 3% BSA for 1 h. Incubation with primary antibodies (diluted 1: 50; Cell signaling Technology) against NM IIA and (diluted 1: 50; Santa Cruz) against EGFR were carried out overnight at 4°C. Second antibodies (Alexa Fluor® 488 dye and Alexa Fluor® 594 dye (Life Technologies)) were incubated 1 h, and then coverslipped with ProLong Gold anti-fade with DAPI (Life Technologies) 5 min before imaging. A laser scanning confocal microscope FV10-ASW (Ver 2.1, MPE FV1000; Olympus Corp.) was used for imagining.

Immunohistochemical analysis for apoptosis, cleaved-caspase 3, p-ERK, Cav-1, EGFR and NM IIA in tumor tissues

Apoptosis in tumor tissues was examined by immunohistochemistry using the *In situ* Cell Death Detection kit (Roche Diagnostic). Paraffin-embedded tissue sections were deparaffinized in xylene followed by treatment with a graded series of alcohol [100%, 95%, and 80% (pH 7.5). Antigen retrieval for ethanol/double-distilled H_2O (v/v)] and rehydrated in PBS paraffin-embedded tissues was performed with sodium citrate 0.01 mol/L (pH 6.0) for 98°C for 5 min. Endogenous peroxidase was blocked by the use of 3% hydrogen peroxide in methanol for 10 min. The samples were washed thrice with PBS and incubated for 20 min at room temperature with a protein blocking solution containing 5% normal horse serum and 1% normal goat serum in PBS. Excess blocking solution was drained, and the samples were incubated overnight at 4°C with one of the following: 1: 100 dilution of rabbit polyclonal anti-human cleaved caspase 3 (Cell Signaling Technology), 1: 200 dilution of rabbit polyclonal anti-human p-ERK (Cell Signaling Technology), 1: 50 dilution of anti-Cav-1, 1: 100 dilution of anti-EGFR antibodies, and 1: 50 dilution of anti-NM IIA antibodies. The samples were then rinsed thrice with PBS and incubated for 1 h at room temperature with the appropriate dilution of the secondary antibody. Slides were examined under the microscope. The positive areas were analyzed using by Image-pro plus software analysis.

SUPPLEMENTARY FIGURES AND TABLE

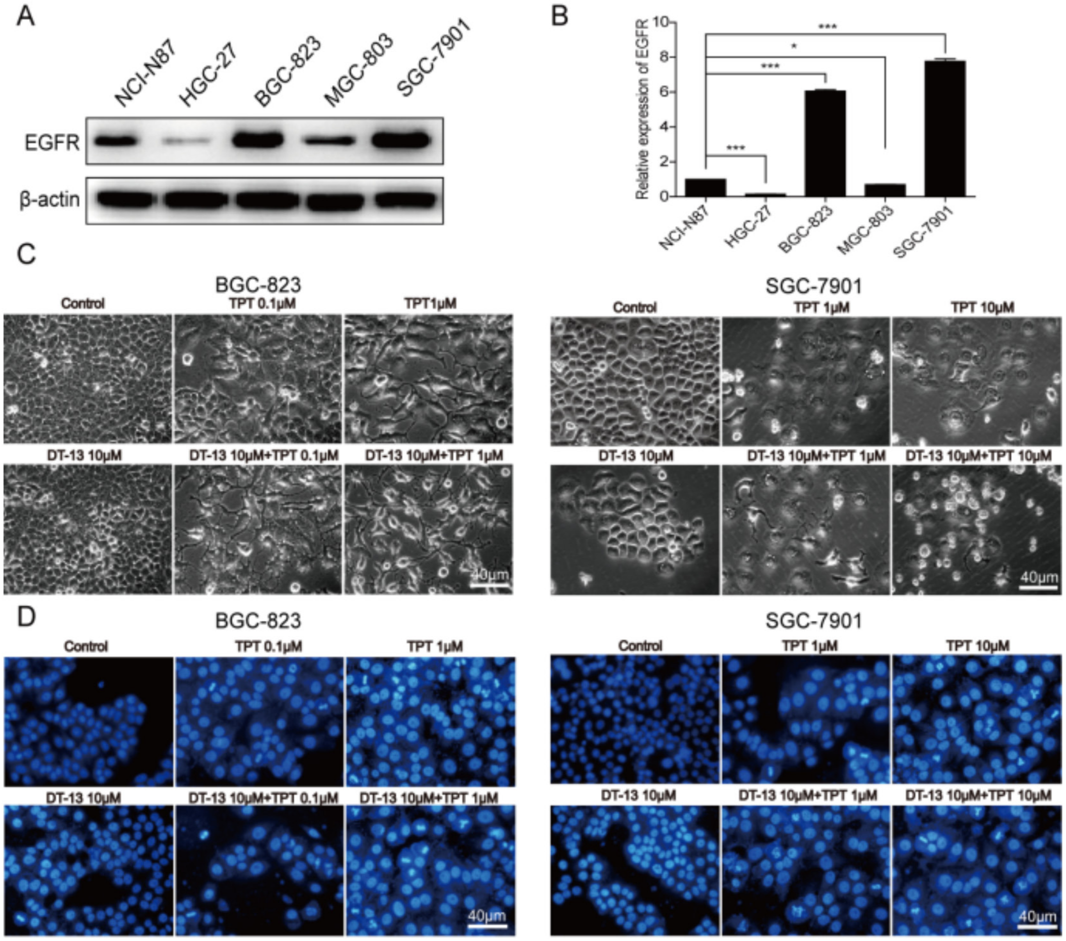

Supplementary Figure S1: The EGFR expression in different gastric cancer cell lines, and the morphological changes caused by DT-13 combined with TPT. A. Western blot analysis of EGFR expression levels in five gastric cancer cell lines. **B.** Quantitative real time PCR analysis of EGFR mRNA levels in five gastric cancer cell lines. After 48 h-treatment, the morphological **C.** and the nucleolus morphological **D.** changes were caused by DT-13 combined with TPT in BGC-823 and SGC-7901 cells. Nucleolus morphological changes were observed by fluorescence microscopy. Statistical analysis was performed using one-way ANOVA followed by Bonferroni's Multiple Comparison Test, *P< 0.05; ***P< 0.001; for B statistical analysis was performed using at least three independent replicates.

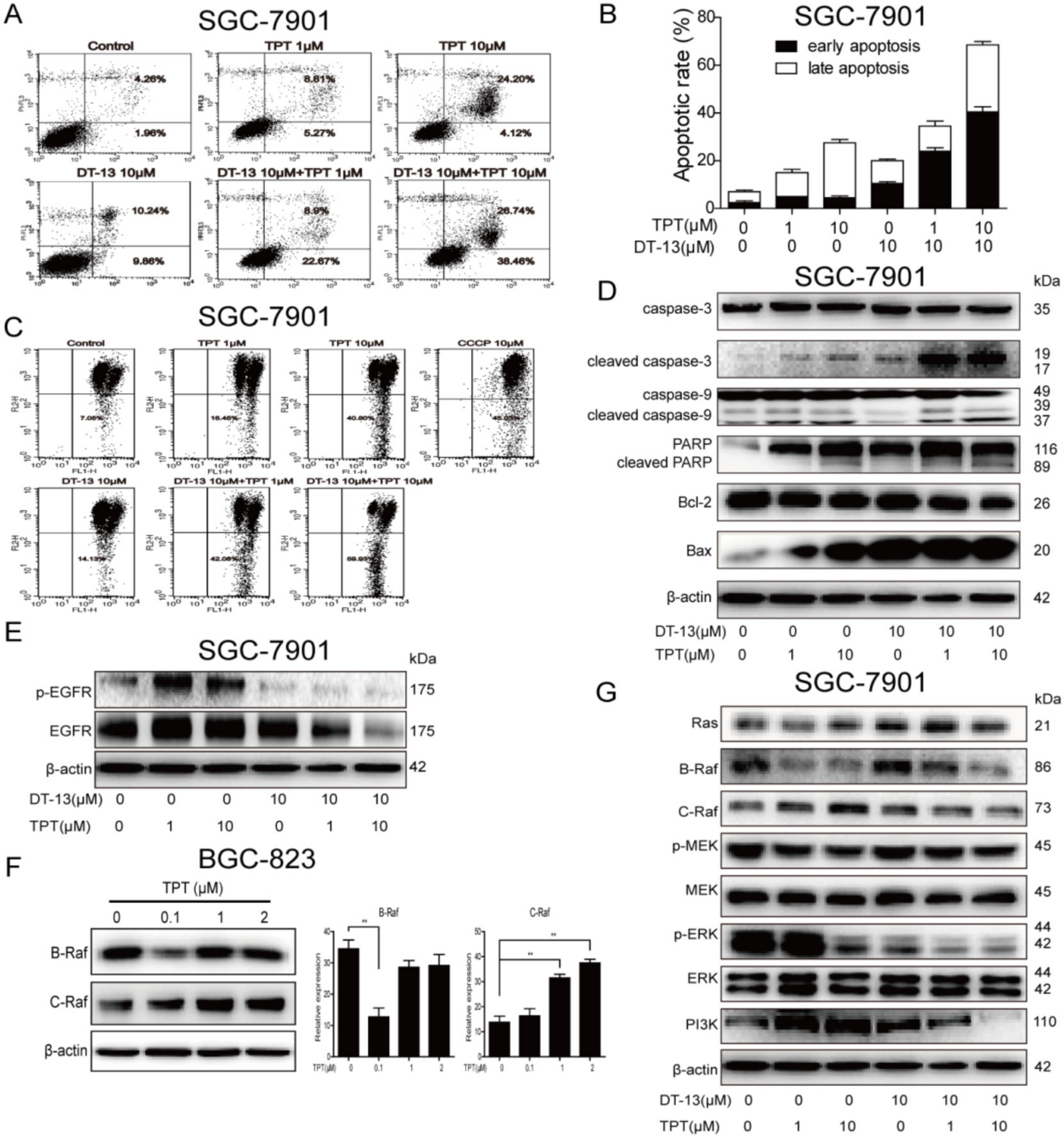

Supplementary Figure S2: DT-13 combined with TPT promoted pro-apoptotic activity and down-regulated EGFR and its downstream signaling in SGC-7901 cells. The cells were measured after the combined treatment for 48 h. The early apoptosis was assessed by Annexin-V/PI staining **A–B.** and JC-1 staining **C.** in SGC-7901 cells. **D.** The protein levels of apoptosis-related proteins were checked by western blotting analysis. **E.** Effects of DT-13 combined with TPT on EGFR total protein levels and phosphorylated protein levels were detected by western blotting analysis. **F.** Effects of TPT mono-therapy on B-Raf and C-Raf protein levels in BGC-823 cells. **G.** Ras/Raf/MEK/ERK1/2 and PI3K signaling pathways in SGC-7901 cells were detected by western blotting analysis. Statistical analysis was performed using one-way ANOVA followed by Bonferroni's Multiple Comparison Test, **P< 0.01.

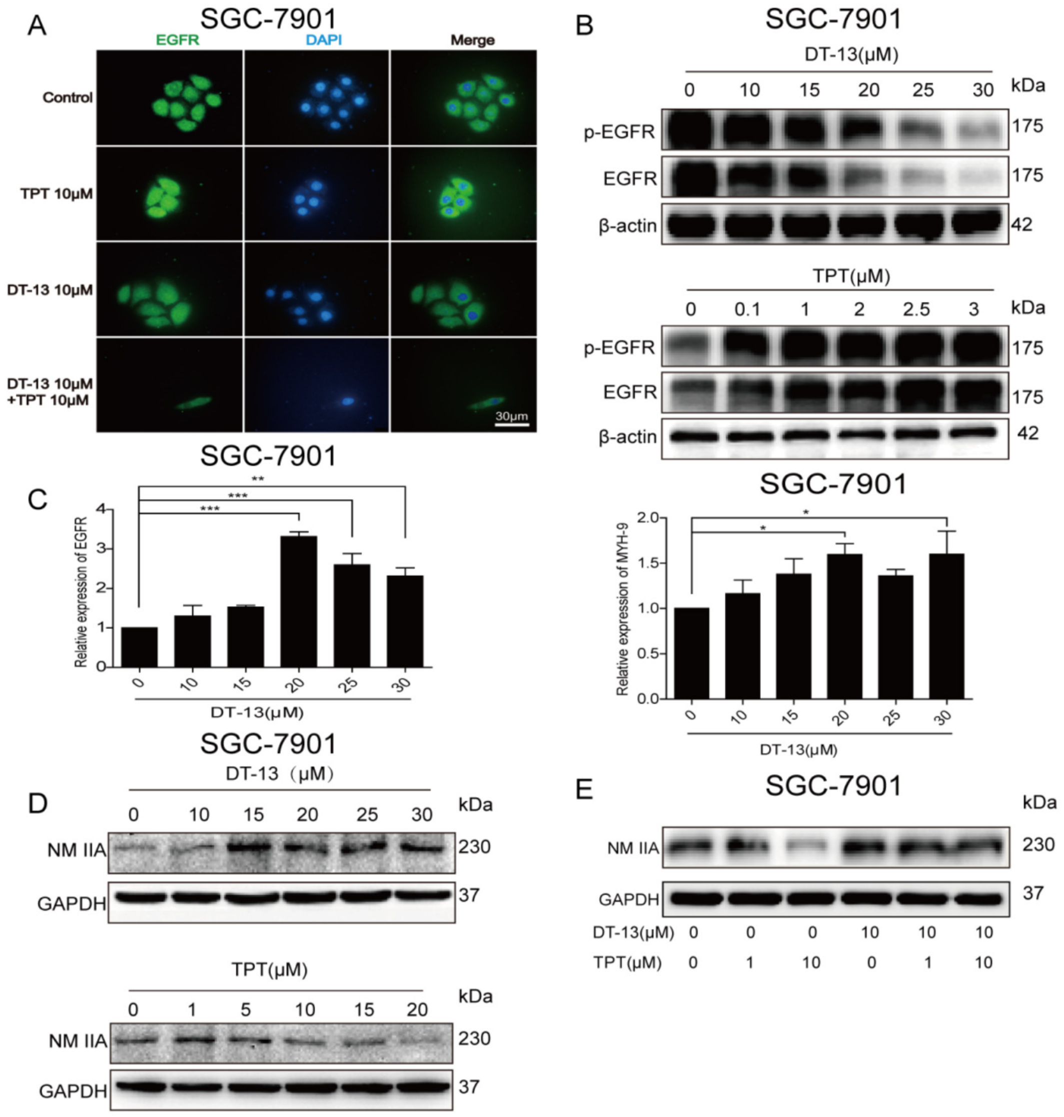

Supplementary Figure S3: Effects of DT-13 and TPT on EGFR and NM IIA in SGC-7901 cells. A. The fluorescence intensity of EGFR was assessed in SGC-7901 cells after the combined treatment for 48 h. **B.** The effect of DT-13 and TPT separately on EGFR and p-EGFR. The cells were treated with DT-13 (10, 15, 20, 25, 30 μM), or TPT (0.1, 1, 2, 2.5, 3.5 μM) for 48 h, the total and phosphorylation levels of EGFR were checked by western blot analysis. **C.** The mRNA levels of EGFR and NM IIA were analyzed by Quantitative real-time PCR analysis when The cells were treated with DT-13 (0, 10, 15, 20, 25, 30 μM) for 48 h. **D.** Western blot assays were used to examine the effect of DT-13 and TPT separately on NM IIA in SGC-7901 cells after cells were treated with DT-13 (10, 15, 20, 25, 30 μM), or TPT (0.1, 1, 2, 2.5, 3.5 μM) for 48 h, GAPDH was used as internal reference. **E.** The expression of NM IIA in SGC-7901 cells was checked by western blot assays after the combined treatment for 48 h. Statistical analysis was performed using one-way ANOVA followed by Bonferroni's Multiple Comparison Test, *P< 0.05; **P< 0.01; ***P< 0.001.

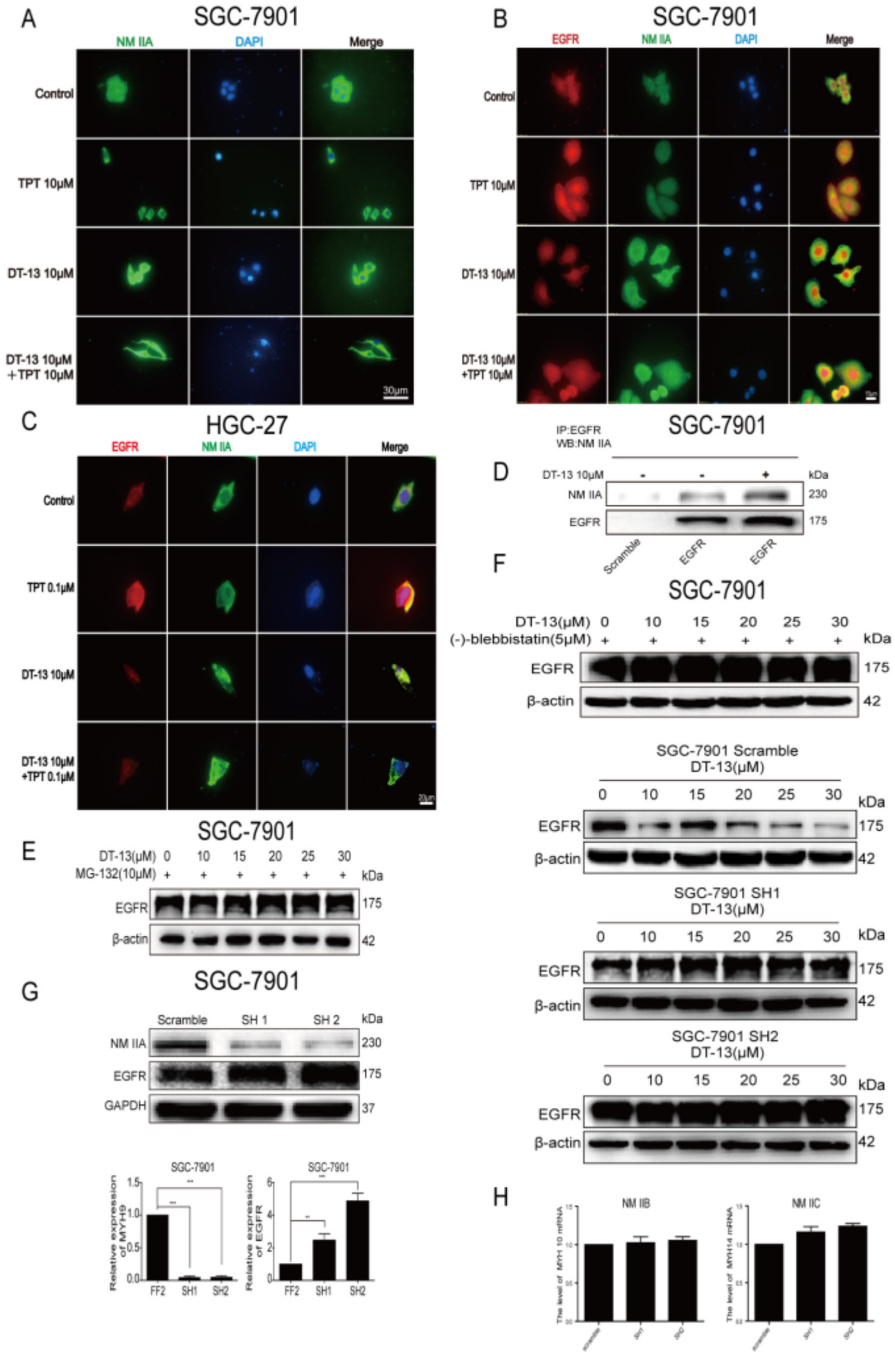

Supplementary Figure S4: DT-13 could promote the degradation of EGFR through endocytosis of EGFR induced by myosin IIA in SGC-7901 cells. A. The immunofluorescence analysis was used to examine NM IIA in SGC-7901 cells when DT-13 combined with TPT. The immunofluorescence localization method was used to verify the relationship of NM IIA and EGFR in SGC-7901 cells **B.** and HGC-27 cells **C. D.** The co-immunoprecipitation assay was used to verify the relationship of NM IIA and EGFR in SGC-7901 cells. **E.** To verify the expression of EGFR protein was ubiquitin mediated protein degradation. The cells were pro-treated with MG-132 for 2 h, and then added DT-13 (0, 10, 15, 20, 25, 30 μM) alone for 48 h. **F.** To verify the degradation of EGFR protein was related to the activity of NM IIA, the cells were pre-treated with NM II inhibitor (-)-blebbistatin or knocked down NM IIA before DT-13 treatment. **G.** After NM IIA knock down, the protein and mRNA levels of EGFR and NM IIA were determined in SGC-7901 cells. **H.** After NM IIA knock down, the mRNA levels of NM IIB and NM IIC were analyzed by Quantitative real-time PCR analysis. Statistical analysis was performed using one-way ANOVA followed by Bonferroni's Multiple Comparison Test, **P< 0.01; ***P< 0.001.

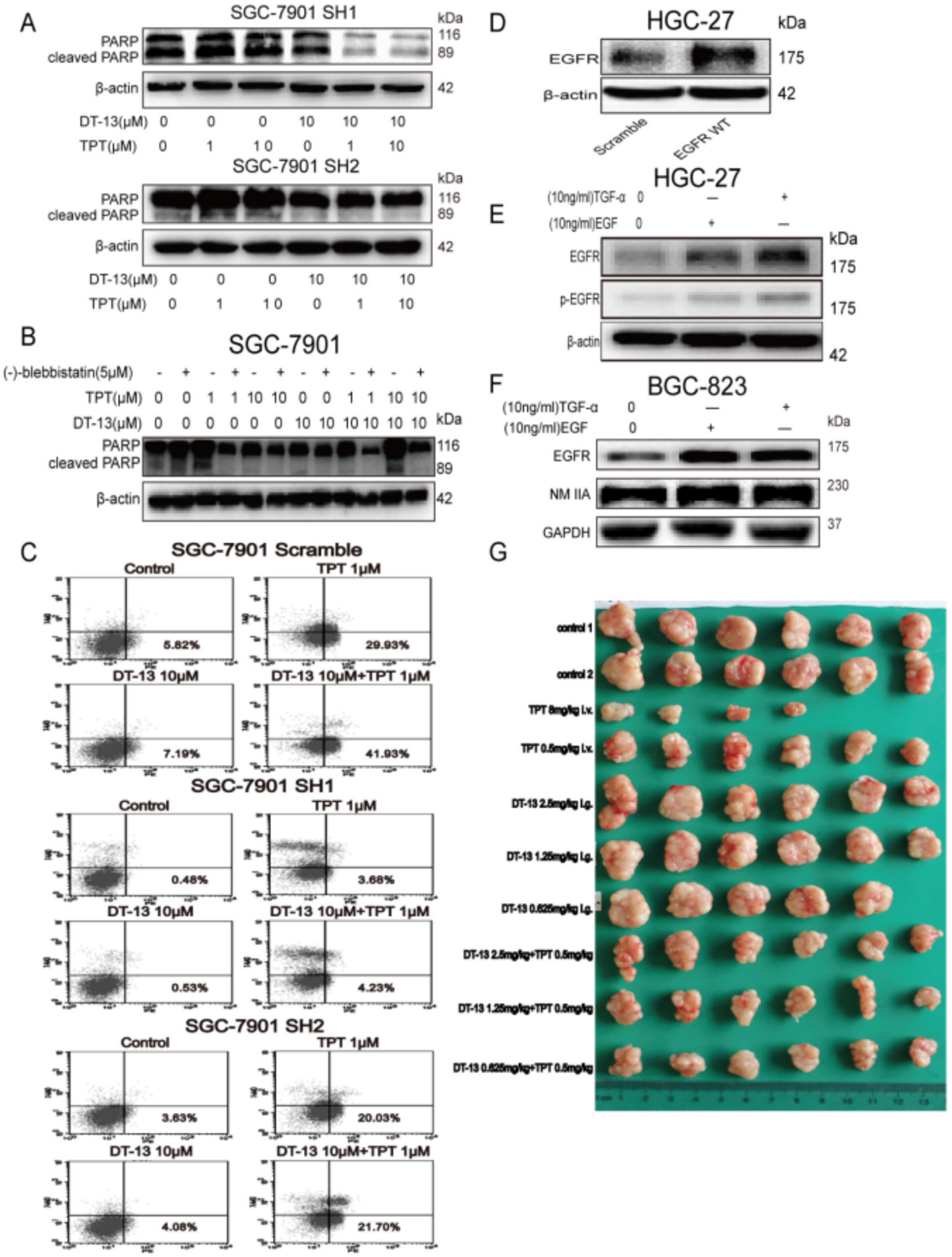

Supplementary Figure S5: DT-13 targeted NM IIA to contribute to the pro-apoptotic effect when combined with TPT. The level of cleaved PARP induced by the combination treatment was reduced by NM IIA knock down **A.** and (-)-blebbistatin **B.** in SGC-7901 cells. **C.** The NM IIA knock down SGC-7901 cells were used to determine the effects of NM IIA on the combination pro-apoptotic activity through Annexin V/PI staining assay. **D.** The EGFR protein level was examined by western blot analysis in HGC-27 cells transfected with EGFR WT plasmid. **E.** The protein level of EGFR and p-EGFR were examined by western blot analysis in HGC-27 cells when treated with EGF (10 ng/mL), TGF-α (10 ng/mL) for 48 h. **F.** Targeting NM IIA prior to EGFR when DT-13-TPT combination. BGC-823 cells were treated with EGF (10 ng/mL) or TGF-α (10 ng/mL), the protein level of NM IIA and total EGFR were checked by western blot assays. **G.** The tumors photographs of BGC-823 cell xenograft model.

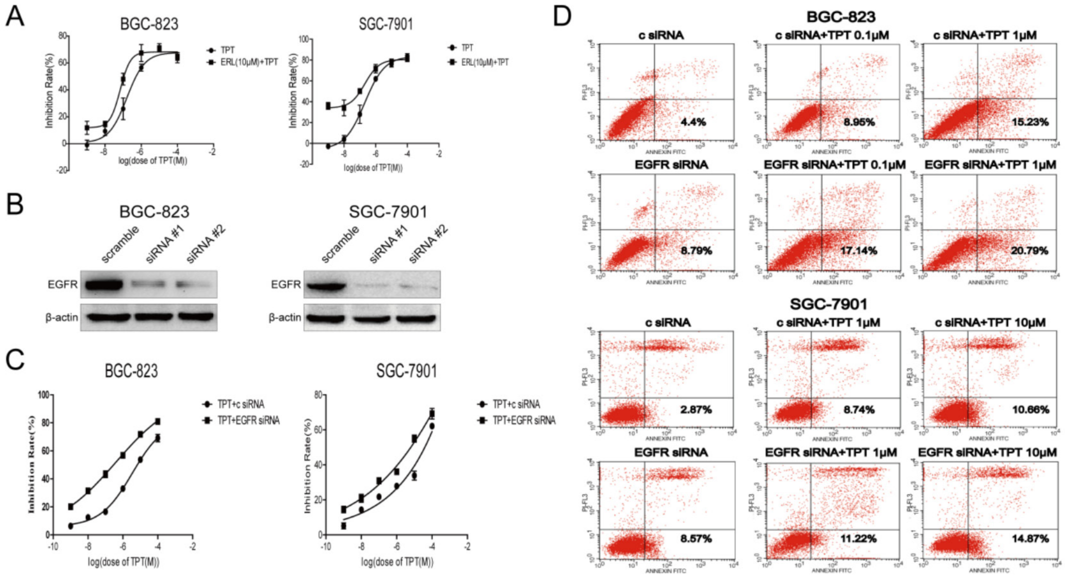

Supplementary Figure S6: The role of EGFR signaling axis in sensitizing gastric cancer cells with high EGFR expression to TPT treatment. A. The combination inhibitory effects of Erlotinib combined with TPT on the survival of GCs (BGC-823, SGC-7901) were measured by MTT assay after the combined treatment for 72 h. **B.** The EGFR protein level was examined by western blot analysis in BGC-823 and SGC-7901 cells when transfected with EGFR siRNA. **C.** The combination inhibitory effects of EGFR siRNA combined with TPT on the survival of GCs (BGC-823, SGC-7901) were measured by MTT assay after the combined treatment for 72 h. **D.** The early apoptosis was assessed by Annexin-V/PI staining in BGC-823 and SGC-7901 cells when treated with EGFR siRNA and TPT.

Supplementary Table S1: Primer sequences for quantitative RT-PCR

Gene	Species	Forward primer (5' to 3')	Reverse primer (5' to 3')
EGFR	Human	TAACGGAATAGGTATTGGTGAATT'	CAGAGGAGGAGTATGTGTGAAGG
MYH-9	Human	ATCCTGGAGGACCAGAACTGCA	GGCGAGGCTCTTAGATTTCTCC
MYH-10	Human	AATAGAGAGACCTGCGAACC	CTTGGAGTGGGAACCTTG
MYH-14	Human	TAGACATTAGGAAGGGAGTGAG	GAGAGGGAGGGAGAGAGAG
GAPDH	Human	CGCTGAGTACGTCGTGGAGTC	GCTGATGATCTTGAGGCTGTTGTC
